# The influence of psychological characteristics on managers efficiency within medical institutions during the COVID-19 pandemic

**DOI:** 10.25122/jml-2022-0295

**Published:** 2023-01

**Authors:** Vasyl Lefterov, Volodymyr Artyomenko, Vasylyna Gutsol, Serhii Harkavets, Larysa Volchenko

**Affiliations:** 1 Department of Psychology, National University Odesa Law Academy, Odesa, Ukraine; 2 Department of Obstetrics and Gynecology, Odesa National Medical University, Odesa, Ukraine; 3 Communal Non-Profit Enterprise Center of Primary Health Care No. 18, Odesa, Ukraine; 4 Department of Psychology and Sociology, Volodymyr Dahl East Ukrainian National University, Severodonetsk, Ukraine

**Keywords:** psychological features, leader, leadership style, efficiency, pandemic

## Abstract

This article examines the theoretical and practical aspects of the activity of medical managers, the social and psychological climate within teams, and interpersonal relations. The goal of the study was to investigate the interpersonal interaction styles and intragroup relations between team members and managers, as well as to determine the impact of managers' psycho-emotional characteristics on their effectiveness during the COVID-19 pandemic. A total of 158 medical workers participated in the study, which was conducted in 2021 using a self-developed questionnaire. The expert evaluation method and standardized psychodiagnostic methods were used. We identified negative factors that affected the management of medical institutions during the pandemic, such as deficiencies in material and economic support, low levels of managerial competence, violation of collegiality and fairness principles during duties and rewards distribution, and deficiencies in manager recruitment. The most psychologically challenging aspects of managing or working in a medical facility during a pandemic include excessive emotional tension and stress, high levels of responsibility, lack of management experience and/or competence in crisis conditions, physical overload, work outside of working hours, and lack of adequate rest. A mini-personality profile of the effective manager of medical institutions in pandemic conditions was developed. One of the psychological regularities of a manager's performance identified is the presence of self-regulative skills in negative emotional states, pronounced activity and energy, mobility, and a strong desire for action.

## INTRODUCTION

The relevance of the research topic is driven by the modern socio-economic and political processes in Ukraine, the country's increasing integration into the world community, and ongoing reforms in management structures. These developments determine the urgent need for a new look at the effectiveness of management activities and require adequate changes.

The issues of management effectiveness in medical institutions were especially relevant during the COVID-19 pandemic, which has presented an unprecedented challenge on a global scale. The pandemic put significant strain on national and international healthcare systems and management structures at regional, state, and transnational levels. The professional, physical and emotional demands on medical workers and medical institutions as a whole significantly increased. Many medical managers found themselves in an unusual situation, "on the front line" of the pandemic, as they worked to implement management tools, crisis management methods, and functions.

The principles of psychological crisis management in special activity conditions have been thoroughly studied in extreme and crisis psychology, particularly in the context of law enforcement agencies, military formations, and rescue services. However, the study of psychological regularities and effective managerial phenomena activity in the medical field, precisely the psychological characteristics and activity of medical institution managers during the pandemic, has not been sufficiently explored. This lack of research highlights the relevance and importance of this research article.

### Recent research and publications analysis

Modern studies on the psychological aspects of management address various topics, including the factors that shape the psychological nature of leadership and management, manager's behavior psychology, the formation of socio-psychological patterns within the management system, the psychological aspects of decision-making, psychological and managerial competence evaluation, conflicts in organizational and management psychology, the adaptation of leaders and staff, and psychological and management counseling.

A review of approaches to management psychology revealed that the attitude towards management is predominantly manipulative, mainly due to the influence of neo-positivist psychology [1–3]. American organizational psychology is characterized by its wide range of methods, many of which focus on specific psychological mechanisms, orientation to the individual, and belonging to the operational management methods group that aims to manage "psychological potential" such as skills and attitudes [1].

Western European psychology is known for its focus on theoretical advancement, which results in a significant emphasis on understanding psychological management problems through theoretical and methodological lenses. This approach is primarily driven by the desire to develop a comprehensive theory for the practical application of social psychology in research [1].

L.M. Karamushka developed concepts related to the psychological foundations of management in educational organizations, including change management and organizational development, psychological and managerial counseling, prevention and overcoming of "professional burnout" syndrome etc [4]. L.E. Orban-Lembryk carried out an authentic analysis of the phenomenology, structure, functions, and methodological foundations of management psychology, investigated the socio-psychological features of management, the psychological features of the individual and the organization in management, as well as various applied problems of managerial activities effectiveness [5]. A meaningful analysis of psychological theories in the context of managerial activity, the psychological leader's personality structure, as well as the personal potential in management problems, psychological health as an effective activity and self-management factor, was carried out by E.M. Kailyuk and H.G. Fesenko [6].

Analyzing the current managerial activity problems in the medical field, D. Mountford and K. Webb point out that the quality of medical care around the world has been increasing for many years, but further transformations are needed, and leaders need to guide this process, including doctors and other medical workers, regardless of whether they hold a managerial position or not [7]. Research confirms that properly organized participation of doctors in the medical institutions' management contributes to improved work results. A study conducted by McKinsey and the London School of Economics showed that in hospitals where doctors actively participated in management, important performance indicators were approximately 50% higher than in other hospitals [8]. According to research in the USA and other countries, successful medical organizations usually pay much attention to the quality of medical care, build close relations between medical and administrative workers, and quickly adopt new work methods [9].

Given its scale, the COVID-19 pandemic became an extremely difficult test for Ukrainian society and humanity. As noted by M.M. Slyusarevsky, in connection with the pandemic, the feeling of unfair and uneven distribution of quarantine-related difficulties gradually grew in society. Existing psycho-emotional tension caused by long-term stress tends to accumulate and can lead to delayed maladaptive reactions deployment to the pandemic, which can entail a complex set of unwanted medical, social and economic risks. These reactions, according to M.M. Slyusarevsky, can develop in several directions, including an increase in anxiety, depression, phobias, post-traumatic stress, and other mental disorders in response to a significant socio-economic deterioration, need for protective and restrictive measures, denial and sabotage, increasing social dissatisfaction and protest moods [10].

In these conditions of social instability, it is the managers who are entrusted with great responsibility. According to T.V. Hura, the leader-manager must maintain positive, warm, trusting relations with others, show autonomy, such as the ability to regulate their behavior and that of their subordinates, and not adhere to collective beliefs, superstitions, fears etc [11].

As T. Gavrish rightly points out, any medical institution requires the introduction of anti-crisis management in difficult conditions. Even successful business management, which is more flexible and prepared for crises than the administrative communal institutions' apparatus, was not ready for the pandemic. It is now almost impossible to hire people to take over running the hospital. Therefore, looking for alternative, fast, and effective mechanisms is necessary [12].

In order to strengthen the mental health strategy during the COVID-19 pandemic, it was necessary to psychologically support the healthcare workers involved in the coronavirus disease outbreak. According to Yu.A. Paskevska, the COVID-19 pandemic posesed certain challenges for the managers of healthcare institutions and requires greater preparedness to preserve the mental health of employees at the individual, managerial and organizational levels. The researcher identified key blocks for managers to meet psychological healthcare needs and proposed an appropriate management model for practical implementation by healthcare institutions managers, integrating doctors, psychiatrists, psychologists, and social workers [13].

This study aimed to examine the socio-psychological characteristics of medical institutions and the personal qualities of their managers. We aimed to investigate the relationship between interpersonal interactions, intragroup relations, and psycho-emotional features of managers and their leadership effectiveness during the COVID-19 pandemic.

## MATERIAL AND METHODS

An empirical study on the psychological characteristics of medical managers and their effectiveness was conducted in 2021 and organized into four consecutive stages:

1. Analysis of previous studies on psychological management effectiveness issues and the peculiarities of medical institutions during the pandemic;
2. Selection of research sample, methods, and valid psychodiagnostic techniques;
3. Organization of all empirical research procedures;
4. Analysis and interpretation of research results and development of practical recommendations to increase the management effectiveness of medical institutions in crisis conditions.

A survey of 158 workers from various medical institutions in Odesa was conducted, including mid-level medical staff, doctors, and directors (chief doctors). The psychodiagnostic study was conducted at the Communal non-profit enterprise Odesa City Council Children's City Clinical Hospital No. 3, which formed the primary sample group. The study included 58 employees and managers, 8 representatives from the hospital management or its divisions, 38 doctors, and 12 representatives from the middle and junior medical staff. The sample was composed of 64% women and 36% men, with the majority of respondents aged between 31 and 45 years and an average age of 41 years.

Additionally, an expert evaluation was conducted to select management representatives who were considered to have a high level of managerial efficiency. These individuals were then subject to psychodiagnostic procedures. The study utilized a combination of self-administered questionnaires, expert evaluations, and standardized psychodiagnostic methods to identify key patterns of effective psychological management. This included managers' self-assessment of communication styles, as well as evaluations by their subordinates.

The questionnaires provided insight into the general socio-psychological management characteristics of medical institutions during the pandemic, as well as an examination of the personal qualities of effective leaders within these institutions. A specially designed form was used by an expert team to evaluate the views of hospital managers. To ensure confidentiality, the data collected were encrypted. Appropriate standardized methods were selected for the psychodiagnostic research [14–18]. Statistical processing was carried out using the Pearson correlation coefficient and Student's t-test.

## RESULTS

65% of the respondents reported that the most psychologically challenging aspect of managing or working in a medical institution during the pandemic was excessive emotional tension and stress (Table 1).

**Table 1:** Psychologically challenging aspects of managing or working in a medical institution during a pandemic.

	Frequency %
**Lack of managerial experience and/or competence to work in crisis conditions**	25
**High responsibility level**	38.7
**Excessive emotional tension and stress**	65
**Distorted information perception**	11.9
**Increased requirements for isolation and limiting contact with others**	16.9
**Strained relations with team members**	5.1
**Conflicts and misunderstandings with patients during their duty's performance**	23.7
**Conflicts with senior management, authorities, media representatives etc**	5.1
**Work outside working hours, lack of full rest**	25.4
**Physical overload**	27.1
**An insecurity and feeling of danger**	22
**Work in extreme mode**	25.4
**Usual work mode rejection**	18.6
**Feelings of increased anxiety or helplessness**	22

A high level of responsibility (38.7%), insufficient managerial experience and/or competence to work in crisis conditions (25%), physical overload (27.1%), working after hours (25.4%), lack of adequate rest (25.4%), work in an extreme mode (25.4%) were also identified as challenging aspects (Table 1).

According to the respondents, the most important factors that facilitate effective management of medical institutions during the pandemic are proper remuneration (79.7%) and coordinated actions, and mutual team support (72.9%). Other factors that were identified as important include managers' experience and competence (67.8%), opportunity to create safe working conditions (66%), high organizational and leadership skills, and management abilities (57.6%) (Table 2).

**Table 2:** Factors contributing to the effective management of medical institutions during the pandemic.

	Frequency %
**Managers experience and competence**	67.8
**Management representatives' high organizational and leadership skills and abilities**	57.6
**High-quality training (special training) for management representatives**	40.7
**Coordinated actions and mutual team support**	72.9
**Trust/friendly relations between team members**	28.8
**Fair duties distribution between team members**	44.1
**Ability to create safe working conditions**	66.1
**High employee motivation**	39
**Adequate remuneration for medical workers**	79.7

According to survey results, 74.6% of respondents cited poor material and financial support as a major factor negatively impacting the management of medical institutions during the pandemic. Other issues identified included a low level of managerial competence (32.2%), errors in planning and organization (32.2%), violations of fairness and collegiality principles in the distribution of duties and rewards (27.1%), and inadequate personnel selection by managers (27%) (Table 3).

**Table 3:** Factors negatively affecting the management of medical institutions during the pandemic.

Answer options	Frequency %
**Recruiting managers disadvantages**	27.1
**Errors in planning and work organization**	32.2
**Low informational and methodological support level for medical institutions' activities during pandemic conditions**	20.3
**Disadvantages in organizational and managerial communication**	18.6
**Ineffective control over the implementation of management decisions**	10.2
**Managers psychological unpreparedness to work in extremely difficult and stressful conditions**	22
**Low level of managerial competence**	32.2
**Material and economic support disadvantages for the medical institutions' activities in the pandemic conditions**	74.6
**Collegiality and fairness principle violation during the duties and rewards distribution**	27.1
**Unhealthy moral and psychological climate in the medical institution staff**	16.9
**Unsatisfactory (non-optimal) medical institution work organization in the pandemic conditions**	30.5

Respondents were asked open-ended questions regarding the psychological difficulties faced while performing their duties in a medical institution during the pandemic. The most common answers are shown in Table 4.

**Table 4:** Psychological difficulties encountered at work.

The risk of contracting the virus for oneself and one's family members, as well as isolation from loved ones who also wish to avoid infection
**Lack of competence and skills, starting with the regional health department**
Increased workload
Moral and psychological burnout
Influx of misinformation and fake news
Delay in examination and patients' diagnosis
The top management orders do not always correspond to reality and possibilities
Situation underestimation by the population
A lot of time spent on the documentation preparation (it is often unclear why), which is also duplicated several times
Lack of professionalism and outright lies in reports
The need to simultaneously perform several types of activities and the understanding that it will not end for a very long time
Overload and lack of time for rest

10.2% of respondents identified the overall management of medical institutions during the pandemic as ineffective, 16.9% considered it satisfactory, 54.2% determined the management as a whole effective, and 18.6% defined the management as very effective.

The suggestions for improving the management of medical institutions during a pandemic include:

1. Providing financial incentives for medical institutions, doctors and managers;
2. Selecting and appointing competent personnel to management positions, promptly certifying and assessing the professional suitability of managers, making changes in management in case of negative performance indicators;
3. Creating an effective headquarters for decision-making;
4. Supporting doctors in providing adequate care to patients;
5. Providing necessary protective equipment, preventive measures, and treatment;
6. Ensuring modern facilities and equipment;
7. Streamlining staffing and documentation;
8. Prioritizing; patient well-being over personal gain;
9. Implementing successful strategies from other countries.

Additionally, participants emphasized the need for empathy and understanding from management and highlighted the most important personal qualities and characteristics of effective managers in a pandemic. For example:

*Stop scolding us constantly, people are falling off their feet from the amount of work, but they only hear that we are not working enough, I suggest managers who think that it is hard to work in our regime for at least a week*.

The survey results on the personal qualities of effective managers have allowed for the identification of key psychological traits and characteristics. According to the factor analysis results, the 15 most notable personality qualities and characteristics of successful managers in a pandemic have been compiled into a mini-profile of an effective medical institution manager, which are presented below:

1. Ability to control emotions during a crisis;
2. The ability to prove and defend one's point of view at any level;
3. Tact, ability to deal with different characters, different social status people;
4. Quick adaptation to new living conditions;
5. High personal discipline;
6. Courage, inclination to reasonable risk in making non-standard decisions and implementing them;
7. The ability to motivate others for effective professional activity;
8. A sense of duty;
9. Organizational abilities;
10. The ability to find the right tone, appropriate behavior when communicating with subordinates;
11. Ability to work conflict-free in a team;
12. Ability to find new unconventional solutions;
13. The ability to resist influence;
14. The ability to foresee and predict the course of future events, taking into account their occurrence probability;
15. Focus on cooperation and teamwork.

Using the "express methodology for the psychological climate study in the working space" in Odesa Children's City Clinical Hospital No. 3, we found that its components are expressed differently:

- Emotional component is positive (0.34);
- The cognitive component (0.21) – contradictory, uncertain;
- The behavioral component is positive (0.33).

Positive ratings, *i.e*., higher than 0.3, were obtained when the number of team members who rated the climate positively exceeded the number of employees who rated it negatively or uncertainly.

The behavioral component includes performance results, actions, facial expressions, gestures, and speech. The emotional component manifests itself in various emotional components. For example, according to positive and negative emotions, conflict presence (intrapersonal, interpersonal, satisfaction with oneself, work, partner etc).

The cognitive component includes all mental processes related to environmental knowledge and oneself (sensation, perception, memory, imagination, thinking etc).

Thus, the superiority of emotional and behavioral components in a given team (positive assessment of these components by the team members' majority) may indicate a generally favorable psychological climate.

**Figure 1:**
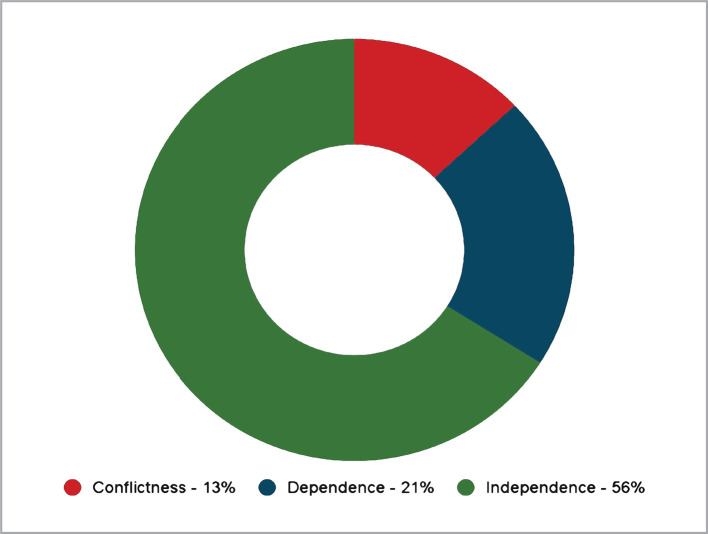
First tendency distribution according to the "Behavior tendencies Q-sorting in the group" method.

The diagram in Figure 1 presents the first percentage ratio using the "Behavior Q-sorting trends in the group" method. From the diagram, it can be seen that the majority of employees are characterized by such a tendency as behavioral independence (66%) in a group.

The tendency to dependence (21%) indicates the individual's desire to accept group standards, defined norms, and values. The desire for independence in the group (13%) indicates the conflict and disunity present within the group. It is also possible to have several small groups within a large group.

Furthermore, we identified that team members were generally sociable and independent, and the tendencies to accept and avoid fighting were equal (49% and 49%). These behavioral prevalence trends confirm this organization's specificity and are the foundation for creating a favorable atmosphere.

The interpersonal relations diagnosis method by T. Leary was carried out to identify the dominant interpersonal style of each person (Table 5).

**Table 5:** General research results using the DMO method by T. Leary.

Interpersonal relations dominant style	The number of persons belonging to this type	% from the total number
**Authoritarian**	6	11
**Selfish**	4	7
**Aggressive**	1	2
**Suspicious**	5	8
**Obeys**	8	13
**Dependent**	10	16
**Benevolent**	9	16
**Altruistic**	15	26

The majority expressed benevolent, dependent and altruistic interpersonal relations styles. This indicates responsibility towards people, delicacy, kindness, sympathy, care, and the ability to encourage and calm others. Other dimensions represent the propensity for cooperation and compromise when solving problems and in conflict situations, the desire to agree with the other's opinion, following the conventions, rules, and "good manners" principles in relations with people, and taking the initiative in achieving the group's goals.

However, some people in the team belong to different types: authoritarian, selfish, and subordinate. Each style complements the other, compensating for the shortcomings.

Thus, with the help of T. Leary's DMO technique, we could identify predominant attitudes. As a result of an expert evaluation analysis by the medical institution employees, it was found that out of the 8 main hospital managers, three managers were the most successful and effective in all parameters. These managers were identified as IM, PS, and BA. It was established that the team most highly evaluated IM – their business qualities assessment significantly exceeded that of other managers, as well as their effectiveness in managing during pandemic conditions. The team also valued business qualities higher than "human" qualities. However, it should be noted that the criteria for evaluating "business" and "personal" qualities are incomparable. Therefore, for his analysis, we will focus on comparing different managers according to one criterion.

In terms of "business" qualities, PS was also rated quite high, whereas BA had the lowest ratings for this parameter. However, both PS and BA had high ratings for their effectiveness in managing during the pandemic. In terms of "human" qualities, as we have already indicated, IM was considered the strongest, followed by BA.

It was noted that the team's perception of effective leaders and managers did not always align. The subordinates did not view all managers as effective in pandemic conditions.

To understand the psychological factors influencing the effectiveness of the managers, the interpersonal communication styles of each manager were analyzed. The results showed that PS had a dominant aggressive and straightforward behavior style in interpersonal relations, while B.A. had a dependent-obedient style and IM had a benevolent (cooperative-conventional) style.

The aggressive behavior of PS was found to be compensation for feelings of inferiority and self-doubt, while the passive behavior of BA was a result of self-doubt and timidity. The behavior of IM was found to be harmonious and adequate for different situations.

Overall, it can be concluded that PS was a professional manager and leader, but his aggressive behavior was a result of personal issues. BA was a compliant and passive individual who relied on PS for leadership. IM was the emotional leader who was well-liked by the team for her calm and friendly demeanor.

The next research stage was the mathematical processing using comparative and correlational analysis. In order to test previous assumptions about the expert managers' evaluation, we compared the average indicators for each criterion between managers. The Student's t-test values for dependent samples and their significance p-levels are given in parentheses. The business qualities of IM were evaluated significantly higher than in PS (t=2.6, p=0.012) and in BA (t=2.89, p=0.005). The average expert assessment value on "human" qualities in IM significantly exceeds the similar parameter in BA (t=2.19, p=0.032). Other differences are statistically insignificant. Thus, the statistical comparison confirmed the preliminary conclusions made above.

Pearson's linear correlation coefficient was used to identify factors influencing managers' effectiveness. The expert's evaluation data and the individual characteristics did not reveal any significant relationships between the evaluations of BA's effectiveness and other indicators. In PS, business efficiency assessment was significantly positively correlated with "unfriendliness" (r=0.266, p=0.049) and negatively to the "Q-sorting" technique "struggle avoidance" (r=-0.353, p=0.008). This suggests that PS's subordinates' perception of his business qualities was inversely related to his willingness to communicate and avoid conflicts. The higher they rated PS's business abilities, the less inclined they were to engage in communication and avoid conflicts. The same results were found in PS's self-assessment using the "Q-sorting" technique, and they are consistent with the characteristics of PS described in this section according to the DMO methodology. In other words, the more similar the subordinate is to the manager, the higher he evaluates his business efficiency. As for the much more popular IM, there were more correlations with its effectiveness assessment. Thus, the business efficiency assessment was negatively related to the friendly expressiveness (r=0.273, p=0.043) and altruistic (r=0.343, p=0.010) interpersonal relations styles and negatively to the selfish style expressiveness (r=-0.293, p=0.030). There was also a significant negative correlation between the propensity to fight and the business performance assessment (r=-0.326, p=0.015). It is noteworthy that this correlation also aligns with IM's characteristics, and the previously mentioned regularity of managers receiving higher evaluations from subordinates is even more pronounced in IM's attitude. It is likely that this correlation occurs because there were more individuals with similar characteristics to IM in the team (as per the group analysis of DMO results, Table 5).

It was also revealed that the higher the team members evaluate the cognitive socio-psychological climate component, the higher they evaluate the human qualities, which additionally confirms the main hypothesis. It was also interesting that only IM had a significant positive relationship with business efficiency estimates (r=0.254, p=0.047). We compared all these facts with the leadership style (Table 6).

**Table 6:** The research results using the "Leadership style" method by A.L. Zhuravlyova.

No.	Manager	Prevailing styles and their diagnostic indicators
**1**	PS	D-К-L (7-4-2)
**2**	IM	К-L-D (6-3-1)
**3**	BA	L-К-D (3-2-2)

**Figure 2:**
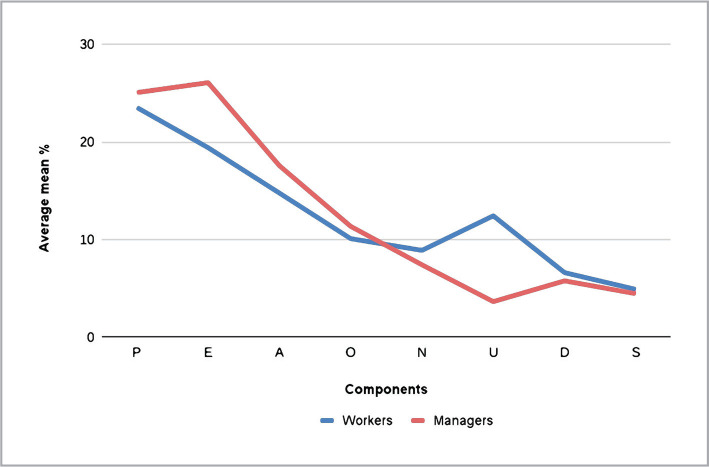
The comparative analysis of generalized psycho-emotional state among employees and effective managers.

The table indicates that the directive management style (D) prevails in PS, with an equal expression of collegiate style in IM (K), and liberal (L) style in BA. However, the management can be characterized as uncertain. We used the Supos-8 method to identify the psycho-emotional characteristics of effective managers by comparing their current and general psycho-emotional state to that of employees (doctors and mid-level medical personnel) (Figure 2). The results showed that the psycho-emotional state of managers differs from that of employees, with managers having higher indicators of mobilizing components and employees having higher indicators of demobilizing components.

When evaluating the statistical significance of differences between the average test values in the experimental and control groups, using the Student's t-test, significant reliable differences (p>0.05) were found for some components. The results of the comparative study on the main psycho-emotional state components are presented in Table 7. As can be seen from the table, the comparative analysis of average values revealed significant differences in the following components (highlighted in the table): component O – impulsive reactivity (in the usual and current psycho-emotional state); component A – desire for action (in a generalized psycho-emotional state).

**Table 7:** The comparative results of the main components of the psycho-emotional state (Supos – 8 methodology).

Scale components	Normal (background) mental state		Current mental state		Generalized test data	
	Difference criterion t	Significance level p	Difference criterion t	Significance level p	Difference criterion t	Significance level p
**Mobilizing components**						
**P – mental peace**	0.7826	0.512	0.3333	0.951	0.3546	0.629
**E – strength and energy**	0.0072	0.680	0.0255	0.222	0.0005	0.217
**A – desire for action**	0.3633	0.161	0.0232	0.544	0.0161	**0.038**
**O – impulsiveness, reactivity**	0.8177	**0.022**	0.4555	**0.001**	0.4375	0.355
**Demobilizing components**						
**N – mental anxiety**	0.6005	0.702	0.1468	0.765	0.1592	0.839
**U – fear, apprehension**	0.0023	**0.019**	2.773	**0.005**	1.6633	**0.001**
**D – depression, apathy**	0.4722	0.310	0.707	0.323	0.4301	0.024
**S – depression, lethargy**	0.6932	0.617	0.2773	0.208	0.6666	0.387

The most likely significant differences (in the usual, current and generalized psycho-emotional state) were found in the component U – fear, anxiety, apprehension. All this indicates that most successful managers have more developed skills for regulating negative emotions, which allows them to reduce fear, anxiety, and apprehension. Additionally, these managers exhibit more pronounced activity and energy, mobility and desire for action, as well as a more confident demeanor.

Based on the findings of our research, which included both quantitative and qualitative analysis, the following was established:

1. The psychological characteristics of a manager play a crucial role in their professional activity basis;
2. In our study, employees rated one manager (IM) whose personal characteristics were most similar to the majority of subordinates' characteristics as the most effective;
3. The most popular leadership style is collegial, which also corresponds to the interpersonal communication dominant styles (benevolent and altruistic) among subordinates;
4. In less effective and less popular leaders, the business and human qualities are not correlated, whereas in effective leaders, they are positively correlated;
5. The influence of personal characteristics on the efficiency evaluation of popular leaders is stronger compared to less popular leaders, and it was not observed in the case of the least popular managers;
6. In contrast to people who do not hold management positions, managers whose activities are evaluated as effective have more developed skills for regulating negative emotions, more pronounced activity and energy, mobility and desire for action, as well as more confident behavior.

## DISCUSSION

People management at any level and in any organization is closely connected to various internal and external factors such as legal, spiritual-cultural, socio-economic, political, ideological, and other human activity spheres. This is especially relevant in modern times, as the COVID-19 pandemic highlighted the need for efficient management in healthcare systems. To effectively respond to this crisis, medical institutions need to quickly reorganize and find effective management solutions [19, 20].

Our study identified several factors that negatively impact the management of medical institutions during the pandemic. These include inadequate material and financial support, low managerial competence, poor planning and organization, violations of fairness principles concerning duties and rewards distribution, and deficiencies in the recruitment of managers.

Furthermore, our results revealed that medical professionals and managers experienced significant psychological challenges during the pandemic, such as high levels of emotional tension, stress, and responsibility, lack of management experience and/or competence to work in crisis conditions, physical overload, work outside regular hours, lack of adequate rest, and work in extreme mode.

Consequently, we were able to develop a mini-personality profile for an effective medical manager in pandemic conditions. The main leadership qualities include the ability to control emotions in crises, strong communication and negotiation skills, tact and adaptability, high personal discipline, courage, a willingness to make non-standard decisions and implement them, and the ability to motivate others regarding effective professional activity.

## CONCLUSION

The psychological traits of a manager play a crucial role in their professional activity basis. Team leadership is associated with interpersonal socio-psychological climate components, in which the leader's human qualities are highly valued. A manager who adopts a collegial leadership style and displays dominant interpersonal benevolent and altruistic communication styles can foster a positive socio-psychological climate and improve production indicators, even if they do not hold formal leadership positions. Moreover, such a personality could compensate for an informal leader, incomplete conformity, and the non-constructive management style of the formal one.

In contrast, less effective and popular managers often have a disconnect between their business and human qualities, but for an effective manager, they are positively related. Popular leaders have a greater impact on the performance evaluation of their subordinates than less popular leaders, and the least popular studied leaders had no influence.

To a certain extent, the dynamics between team members determine the management tactics and strategy. Effective managers have positive traits, such as good emotional regulation, developed skills, expressed activity and energy, mobility and desire for action, as well as more confident behavior.

Future research should focus on practical ways to increase management effectiveness in medical institutions by introducing psychological anti-crisis management training for managers and improving the qualifications system.

## Data Availability

Further data is available from the corresponding author on reasonable request.

## Personal thanks

Our team would like to thank LLC Artemis LTD (Ukraine) for their support in manuscript interpretation and accompaniment.
